# Assessing the susceptibility of schools to flood events in Iran

**DOI:** 10.1038/s41598-020-75291-3

**Published:** 2020-10-22

**Authors:** Saleh Yousefi, Hamid Reza Pourghasemi, Sayed Naeim Emami, Omid Rahmati, Shahla Tavangar, Soheila Pouyan, John P. Tiefenbacher, Shahbaz Shamsoddini, Mohammad Nekoeimehr

**Affiliations:** 1 Soil Conservation and Watershed Management Research Department, Chaharmahal and Bakhtiari Agricultural and Natural Resources Research and Education Center, AREEO, Shahrekord, Iran; 2 Department of Natural Resources and Environmental Engineering, College of Agriculture, Shiraz University, Shiraz, Iran; 3 Soil Conservation and Watershed Management Research Department, Kurdistan Agricultural and Natural Resources Research and Education Center, AREEO, Sanandaj, Iran; 4 Department of Watershed Management Engineering, College of Natural Resources, Tarbiat Modares University, Tehran, Iran; 5 Department of Geography, Texas State University, San Marcos, TX USA

**Keywords:** Environmental sciences, Natural hazards

## Abstract

Catastrophic floods cause deaths, injuries, and property damages in communities around the world. The losses can be worse among those who are more vulnerable to exposure and this can be enhanced by communities’ vulnerabilities. People in undeveloped and developing countries, like Iran, are more vulnerable and may be more exposed to flood hazards. In this study we investigate the vulnerabilities of 1622 schools to flood hazard in Chaharmahal and Bakhtiari Province, Iran. We used four machine learning models to produce flood susceptibility maps. The analytic hierarchy process method was enhanced with distance from schools to create a school-focused flood-risk map. The results indicate that 492 rural schools and 147 urban schools are in very high-risk locations. Furthermore, 54% of rural students and 8% of urban students study schools in locations of very high flood risk. The situation should be examined very closely and mitigating actions are urgently needed.

## Introduction

Floods are among the destructive natural hazards. These extreme events can be generated by a number of natural processes or from human activities and catastrophes, including heavy precipitation events, melting snowpack, modified drainage networks, failures of dams, and manipulation of drainage features. Based on recorded data, floods have caused US $700 billion globally and about 7 million deaths since 1900^1^. Floods are about 30% of hazardous events^2,3^. During last few decades, urbanization and increasing in populations have greatly increased exposure of people and properties to floods^4–6^. Some studies indicate that flood frequency and severity may increase as a consequence of global warming and changing climates^7,8^.

Floods could be managed and mitigated by soft (nature-based and/or non-structural) and hard (engineered and structural) actions and decisions. The hard actions include dams, diversions, and check dams. Soft actions include land use planning, river restoration, selective siting of buildings, flood prediction modeling, alarm systems, improving public awareness of flood hazards, and education^9,10^. Floods influence soil erosion, enhance natural habitats, support ecological processes, and are important to many aspects of human life.

In communities, children are the most vulnerable to the consequences of flood exposure^11,12^. Schools are settings that concentrate children and need special attention with regard to extreme natural events. Over the last few decades, the frequency of floods has been increasing and loss of life and property has accordingly increased^13–15^. So, it is important to assess susceptibility of schools to flood events to reduce damages and prevent loss of lives. For this purpose, a flood susceptibility and hazard map can be prepared using various techniques or algorithms including statistical and machine learning.

Machine learning (ML) algorithms like logistic regressions^16–18^, random forests^19,20^, support vector machines^21–24^, decision trees^25,26^, artificial neural networks^27,28^, boosted regression trees^29–31^, multivariate adaptive regression splines^29,32^, and model-driven architectures^16,33^ have been tested for hazard analysis and mapping in literature. The ML approach has been used to evaluate the risk and susceptibility of communities exposed to a number of extreme and hazardous conditions: landslides^34–36^, wildfires^37,38^, gully erosion processes^39–41^, land subsidence^42,43^, earthquakes^4,13,44^, dust storms^45^, and floods^6,7,46^. Flood-hazard vulnerability has been examined by a number of scholars. Ochola et al. ^47^ studied the susceptibility of schools to floods in the Nyando River basin in Kenya. They analyzed the conditions of 130 schools in the western part of that country and found that 40% were vulnerable to floods. Karmakar et al.^48^ conducted a risk-susceptibility analysis of floods in southwestern Ontario, Canada. They evaluated four types of vulnerability—physical, economic, infrastructural, and social—using a geographic information system (GIS). Balica et al.^49^ examined flood susceptibility using parametric and physical models and concluded that parametric modeling has limited accuracy, but provides a simplified view of social indicators of vulnerability. Nabegu^50^ studied the vulnerabilities of households to flooding in Kano, Nigeria. They found that houses in the most vulnerable zone were destroyed and 17 people lost their lives during flood events. Eini et al.^51^ investigated urban flood susceptibility using ML techniques in Kermanshah, Iran. They prepared flood maps using two ML models—maximum entropy and genetic algorithm—and found that maximum entropy yielded a more accurate flood-susceptibility model. They also determined that infrastructural characteristics had the greatest influence on flood susceptibility. Tascón-González et al.^52^ studied social flood-vulnerability in Ponferrada, Spain using analytic hierarchy process (AHP) and found that 34,941 residents were impacted by floods from a dam break, and that 77% of them suffered heavy damages.

Few have attempted to examine the susceptibility of school locations to floods. A risk assessment of schools in developing countries is very important but has not yet been conducted. This study is the first to investigate the exposure of both urban and rural schools to flood hazards. It has been conducted for the mountainous province of Chaharmahal and Bakhtiari, Iran. The goal is to identify the locations most in need of mitigation to reduce damages and prevent loss of lives. Four ML models were tested and compared for the tasks of mapping flood hazard and assessing schools’ exposures.

## Materials and methods

### Study area

Chaharmahal and Bakhtiari Province is in southwestern Iran in a region dominated by the Zagros Mountains. Having an average elevation of 2153 m above sea level and a range of elevations from 778 to 4203 m, the province is the highest in Iran. The province covers 16,421 km^2^ and its population is approximately 947,000. Due to the topographical and climatic conditions of the region, floods occur annually throughout the province.

### Methodology

**Figure 1:**
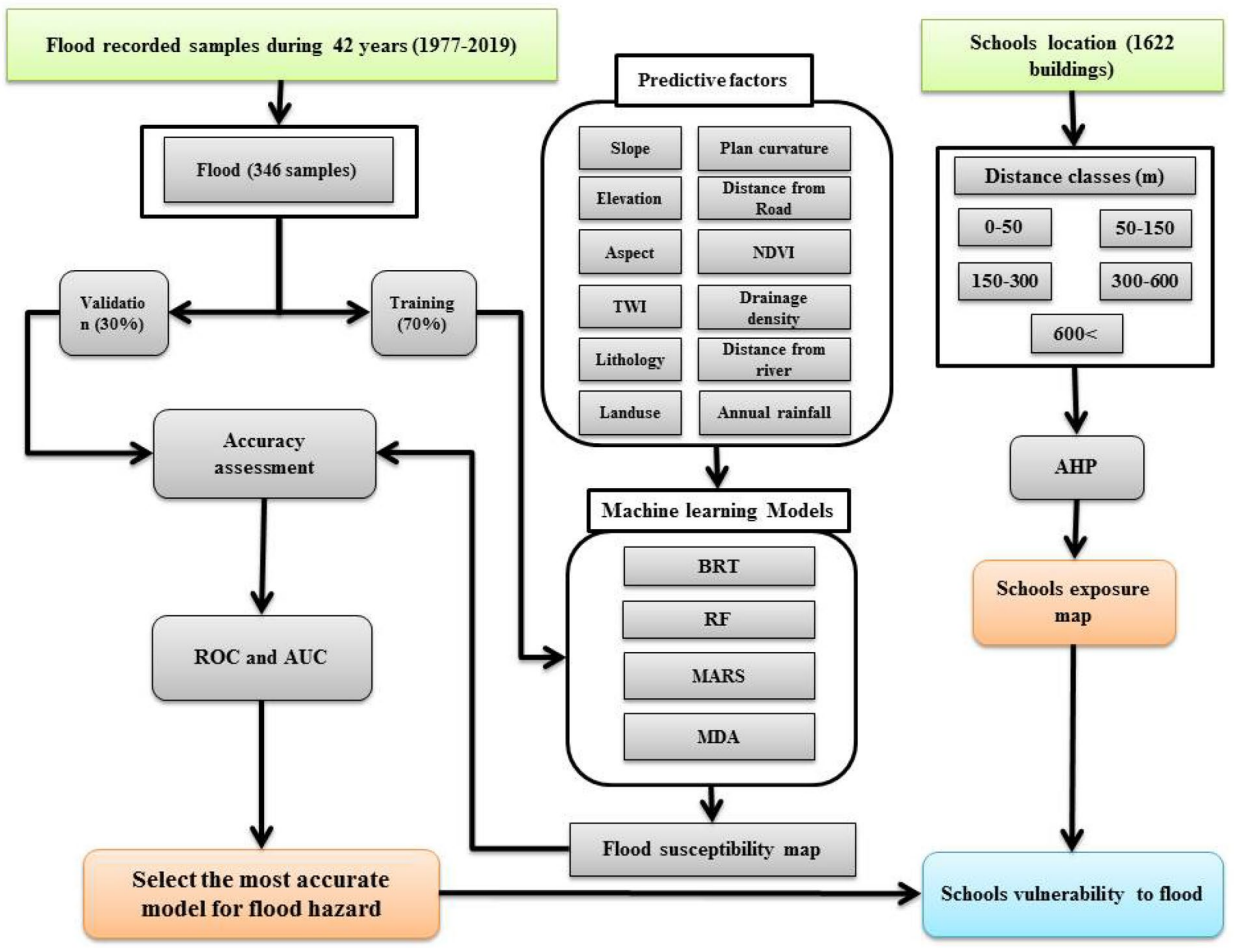
Flowchart of the methodology in present study.

There are five steps to this research: (1) collection and compilation of spatial data; (2) determination of the influence of the independent effective factors on flood probability; (3) production of flood risk maps using four ML algorithms; (4) validation and evaluation of the flood risk maps, and (5) determination of the susceptibility of schools to floods in Chaharmahal and Bakhtiari Province (Fig. 1).

#### Collection and compilation of spatial data

**Figure 2:**
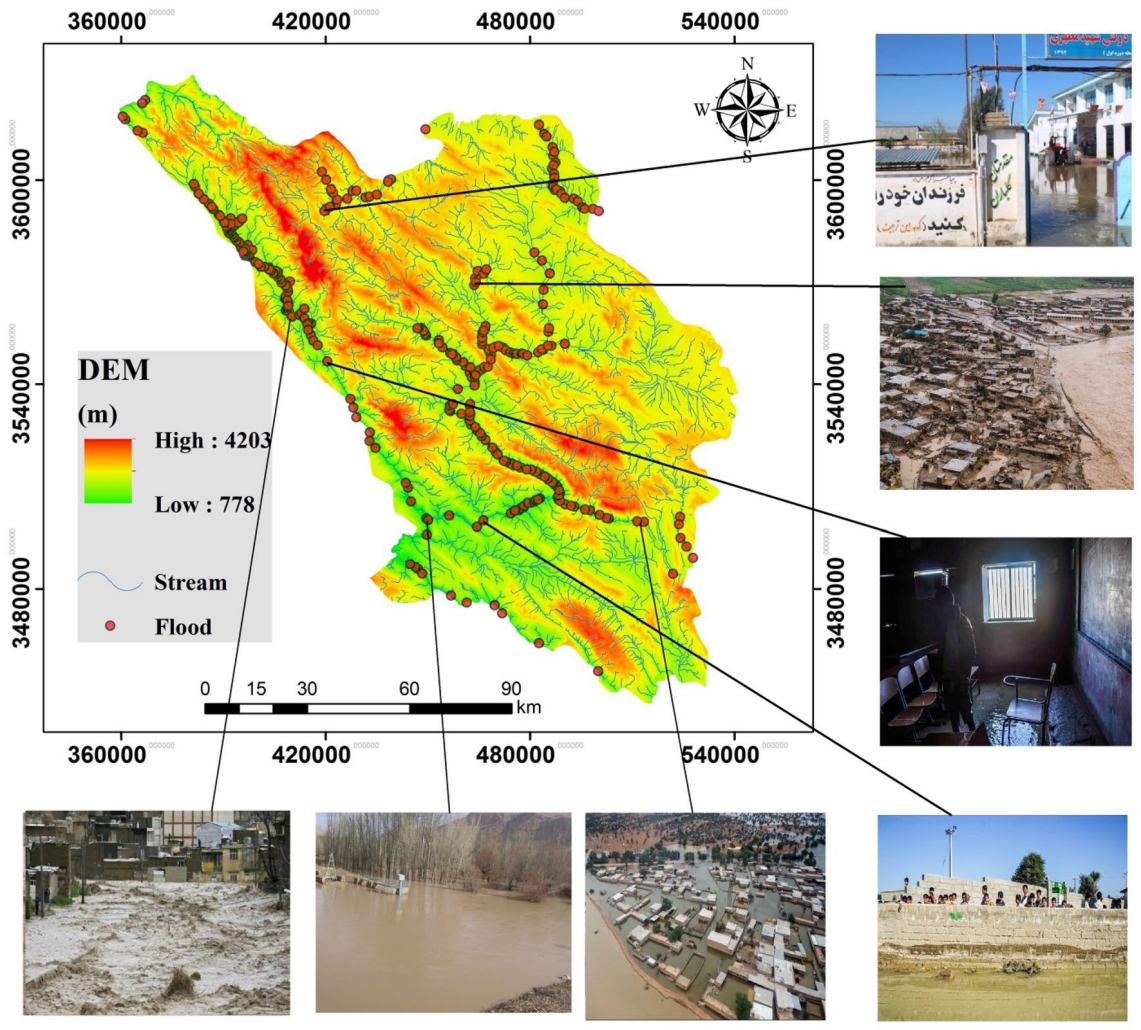
Locations of the floods that occurred between 1977 and 2019 in Chaharmahal and Bakhtiari Province and visual examples of several events.

**Figure 3:**
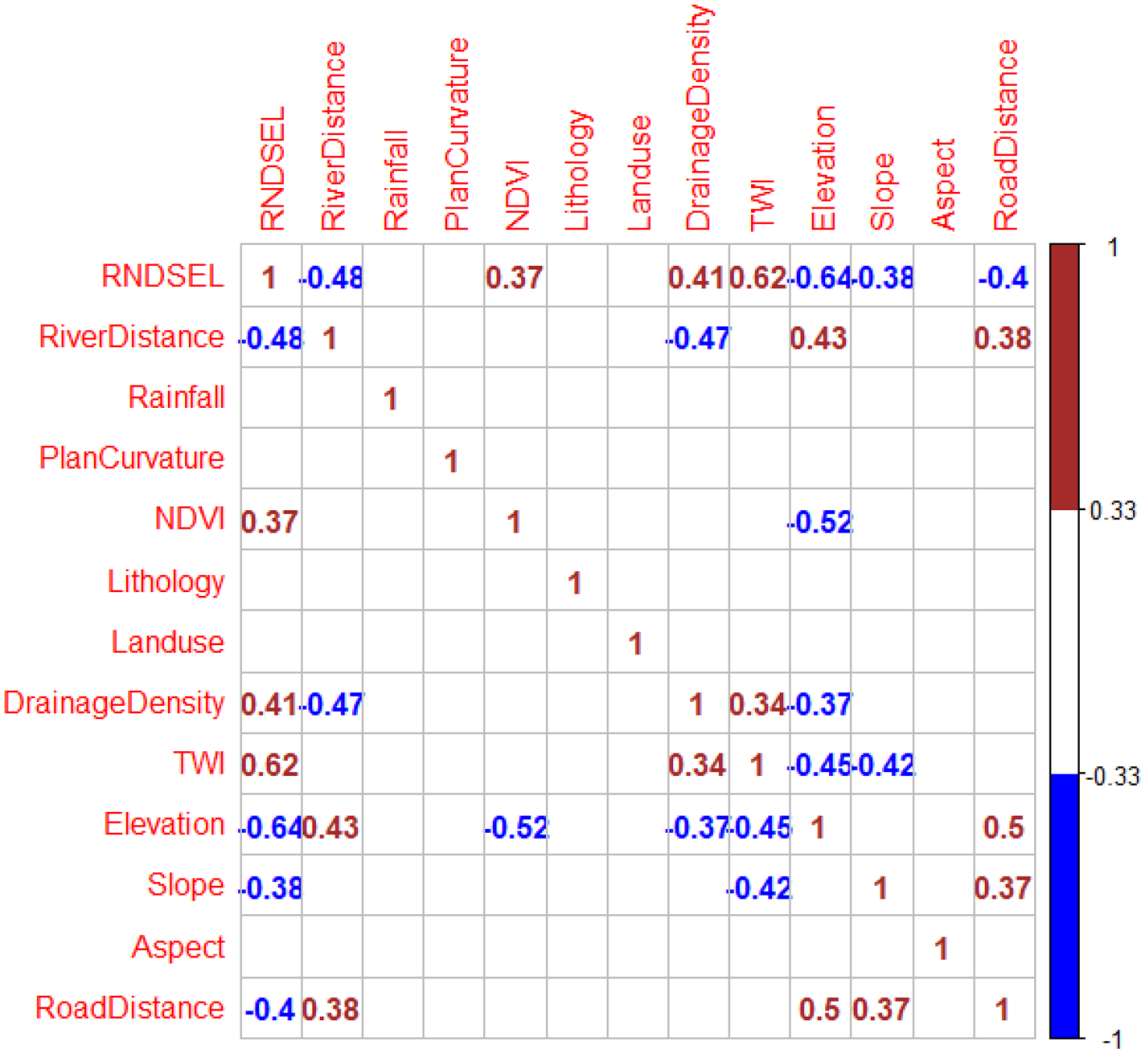
Pearson correlation test among various independent variables (RNDESEL = flood locations).

To accurately determine flood patterns and frequencies in a region, an accurate and well-distributed sample of flood occurrence must be compiled. Three hundred and forty-six floods that occurred in the province were recorded over a 42-year period (1977–2019) by Iran’s Ministry of Energy. The locations of the floods were identified and geo-located using a global position system (GPS) device during extensive field surveys. These points were mapped (Fig. 2). The sample was randomly divided into a modeling set containing 70 percent of the locations and a validation set containing 30% of the sample. As flood occurrence is determined by an interaction of natural and human processes, based on previous studies^15,53–55^ 12 of the most important effective factors were identified for use in modeling as input variables. They included elevation, slope, aspect, plan curvature, lithology, drainage density, annual rainfall, topographic wetness index (TWI), normalized difference vegetation index (NDVI), land use type, distance from nearest river, and distance from nearest road. The data were derived from 1:25,000 topographic maps, 1:100,000 geological maps, and OLI Landsat images (from 2018). The 12 data layers were created in ArcGIS 10.4.2 and ENVI 5.3 software. To ensure that the 12 input factors were truly independent of each other (not highly correlated with each other), a multicollinearity test was applied. The Pearson correlation tests showed no significant correlation between the factors, ensuring a more accurate flood risk map (Fig. 3).

#### Determination of the influence of input factors on flood probability

**Figure 4:**
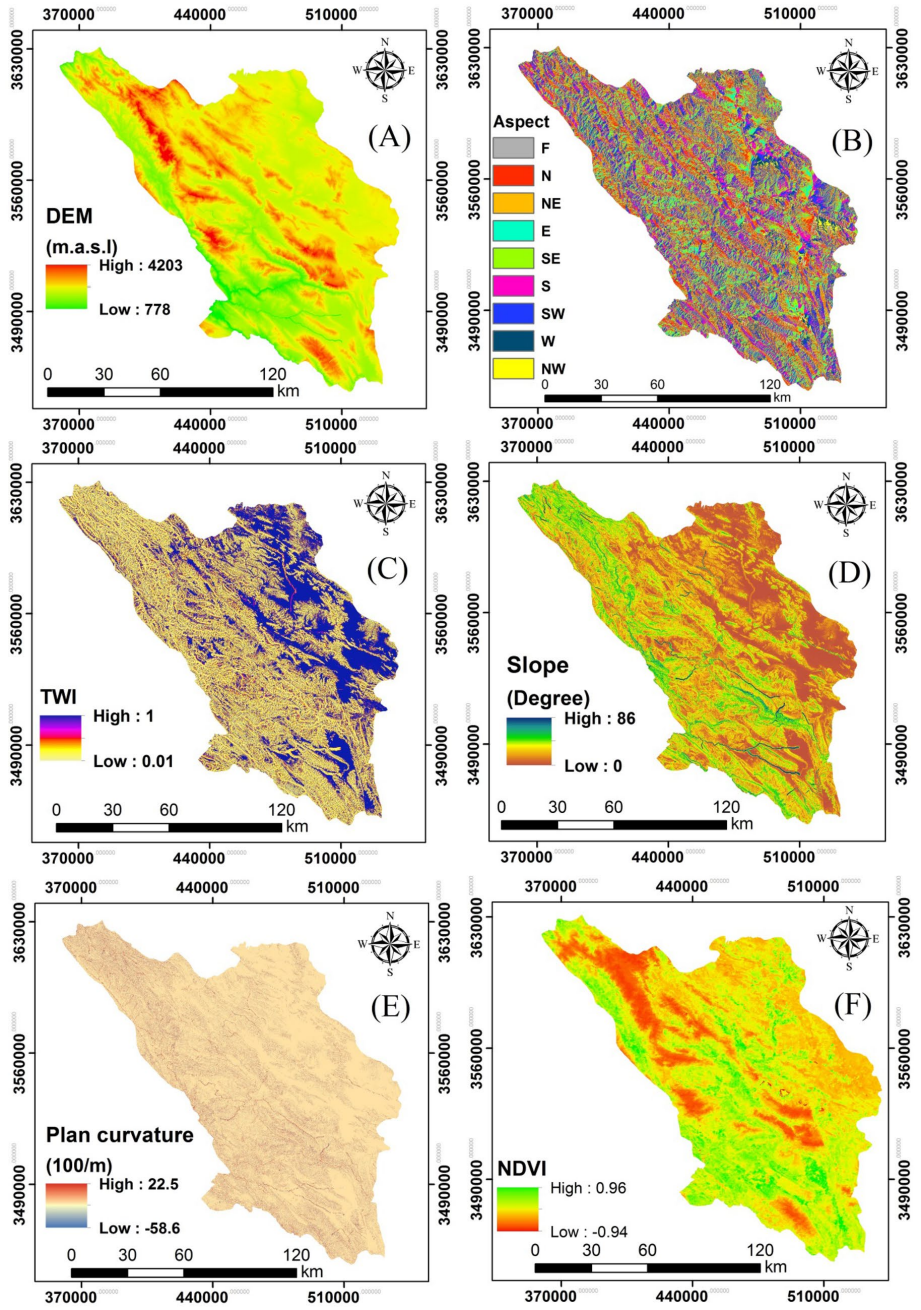

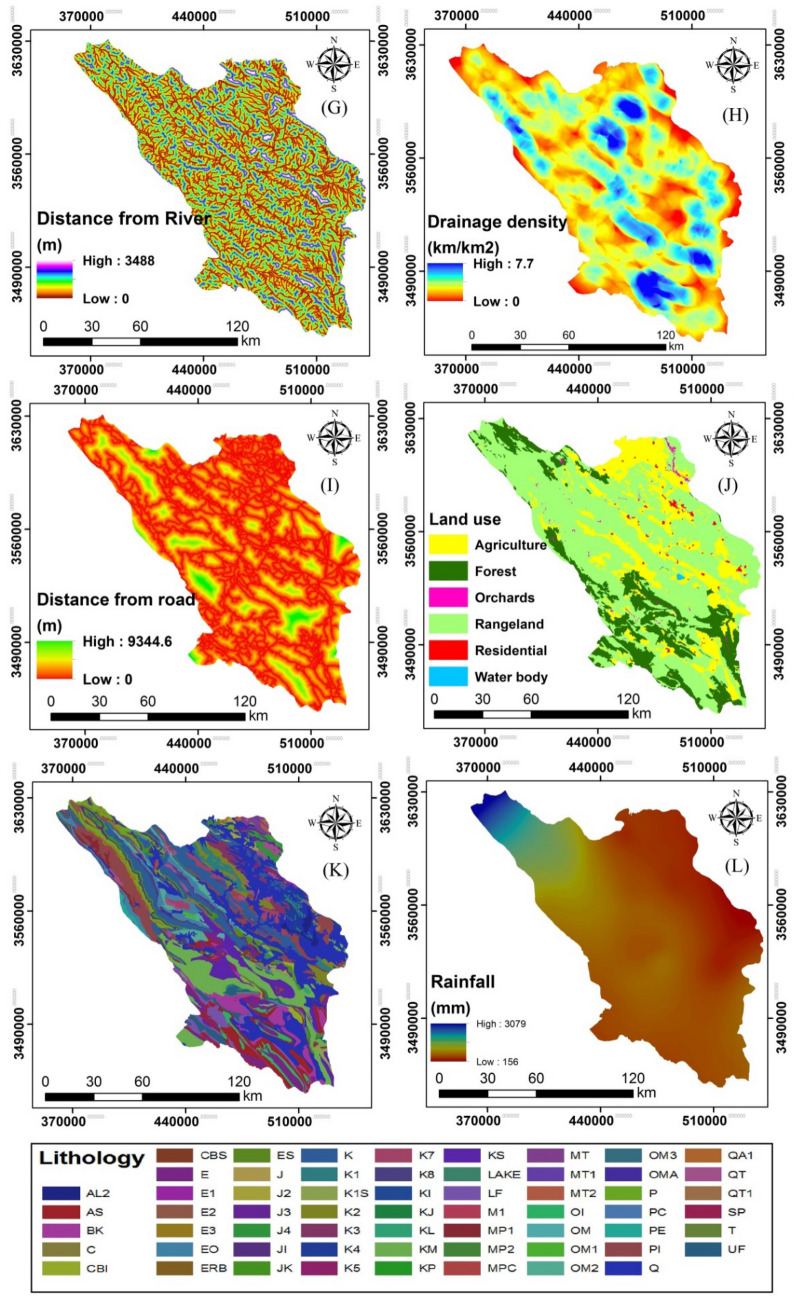
Maps of effective factors in Chaharmahal and Bakhtiari Province: **(A)** elevation, **(B)** aspect, **(C)** TWI, **(D)** slope, **(E)** plan curvature, **(F)** NDVI, **(G)** distance from river, **(H)** drainage density, **(I)** distance from road, **(J)** land use, **(K)** lithology, and **(L)** annual rainfall.

Some topographic factors can interact to increase the likelihood of flooding. Elevation, aspect, TWI, slope, and plan curvature layers were constructed from 1:25,000 topographic maps (Fig. 4A–E). Vegetation is also integral to hydrological processes. An NDVI layer was extracted from OLI Landsat images from 15 Jun 2017 to indicate vegetation patterns (Fig. 4F). The 1:25,000 topographic maps provided streams and road-network information. These were extracted and used to create raster layers of drainage density, distances from rivers, and distances from roads (Fig. 4G–I). The OLI Landsat images were also used to map land uses (Fig. 4J). Lithological units were extracted from a 1:100,000 geological maps acquired from the Iranian Geology Organization (Fig. 4K). Precipitation is a key factor influencing flood occurrence. Data were gathered from 18 weather stations to determine average annual rainfall from 1982 to 2019 and these data were used to reflect the rainfall factor in flood-risk mapping (Fig. 4L).

#### Modeling flood risk using four ML algorithms

##### Boosted regression trees (BRT)

The BRT model is a model that combines methods to improve analysis^56^. Since BRT is usually associated with tree-based methods, it is useful for identification of the factors that most impact predictions of an outcome. A benefit of BRT is that it can work even when some data are absent^36^. BRT balances models’ performances^37,57^ and balances between models’ performances^58^. BRT results are conditioned by the number of trees used in the model and the combinations of the trees used. Performance is improved as the number of trees increases^59^. The following features were set for running the BRT model: gbm.x = 2:13, gbm.y = 1, family = "bernoulli", tree.complexity = 5, learning.rate = 0.005, bag.fraction = 0.5. Here, gbm.x = the 12 independent variables and gbm.y = dependent the variable (flood location). The final BRT model had 1850 trees to predict flood locations. Mean total deviance = 1.386, mean residual deviance = 0.059, estimated cross validation deviance = 0.333, and standard error = 0.055.

##### Mixture discriminant analysis (MDA)

The MDA is a supervised classification algorithm based on mixture models. This model is an extension of linear discriminant analysis and is used to estimate density for each class^60^. In general, the MDA model is suitable for modeling multivariate nonlinearity relationships among various parameters within each group. It is also important to determine whether there are underlying sub-classes in each group which can have a positive effect on the factors of the environment or the independent factors^61–64^. The “mda” package^65^ was used to run the MDA model.

##### Random forest (RF)

RF is a nonparametric technique based on regression trees^20,40,66^. It is one of the strongest ML models due to the large number of trees that it incorporates^67,68^. RF has several advantages: it is insensitive to noise, it can incorporate most types of data, and it is helps to determine the variables that are most important^14,37,69^. Shahabi et al.^70^ indicates that RF is very effective at estimating the relative importance of factors, which aids with decision-making for environmental management. The settings of the RF model were mtry = 4, ntree = 1000, and the estimated out-of-bag (OOB) error rate was 5.27%.

##### Multivariate adaptive regression splines (MARS)

MARS is one of the best regression-based algorithms^13,71^. Its predictions can be made based upon both linear and non-linear relationships between independent factors^72^. This model is very flexible for predicting events based on a set of independent factors. Furthermore, it allows for the determination of the relative importance of the independent variables in the predictions^30,67,71^. MARS determines the relationships between dependent and independent variables and reflects these functions as coefficients so that the impacts of the factors are calculated separately^73^. It defines basic functions by the intervals of the factors^74,75^. MARS has a sensitivity to variable correlations^74^ and has been used in many applications to assess geophysical, climatological, environmental, and geomorphological relationships^76–79^. In this method, pruning was “backward” with three penalties. After pruning, generalized R^2^ was 0.774, whereas R^2^ was 0.824.

##### R statistical packages used for modelling process

The BRT, MARS, MDA, and RF models were run in R software version R 3.5.3. Each required use of specific packages: "brt"^58^, "mda"^61^, "MARS"^74^, and "randomForest"^80^. R software was used to perform the modeling, analysis, and graphical depictions of the analyses^81,82^.

#### Evaluation of the modeled flood-risk maps

The results of the four ML models were evaluated to identify the most accurate model. The receiver operating characteristic (ROC) curve is a cutoff-independent evaluation approach for determining the goodness-of-fit and predictive performance of models. The area under the ROC curve (AUC) was the analysis of accuracy used^83–85^. The validation data set contained 30% of the flood location sample that was not used for training^38,58,86^. The relative importance of each of the independent factors on the modeled flood predictions were analyzed with least absolute shrinkage and selection operator (LASSO). LASSO is a regression-based method that analyses variable selection and regularization in ML models.

#### Determination of the proximity of schools to flood zones

**Figure 5:**
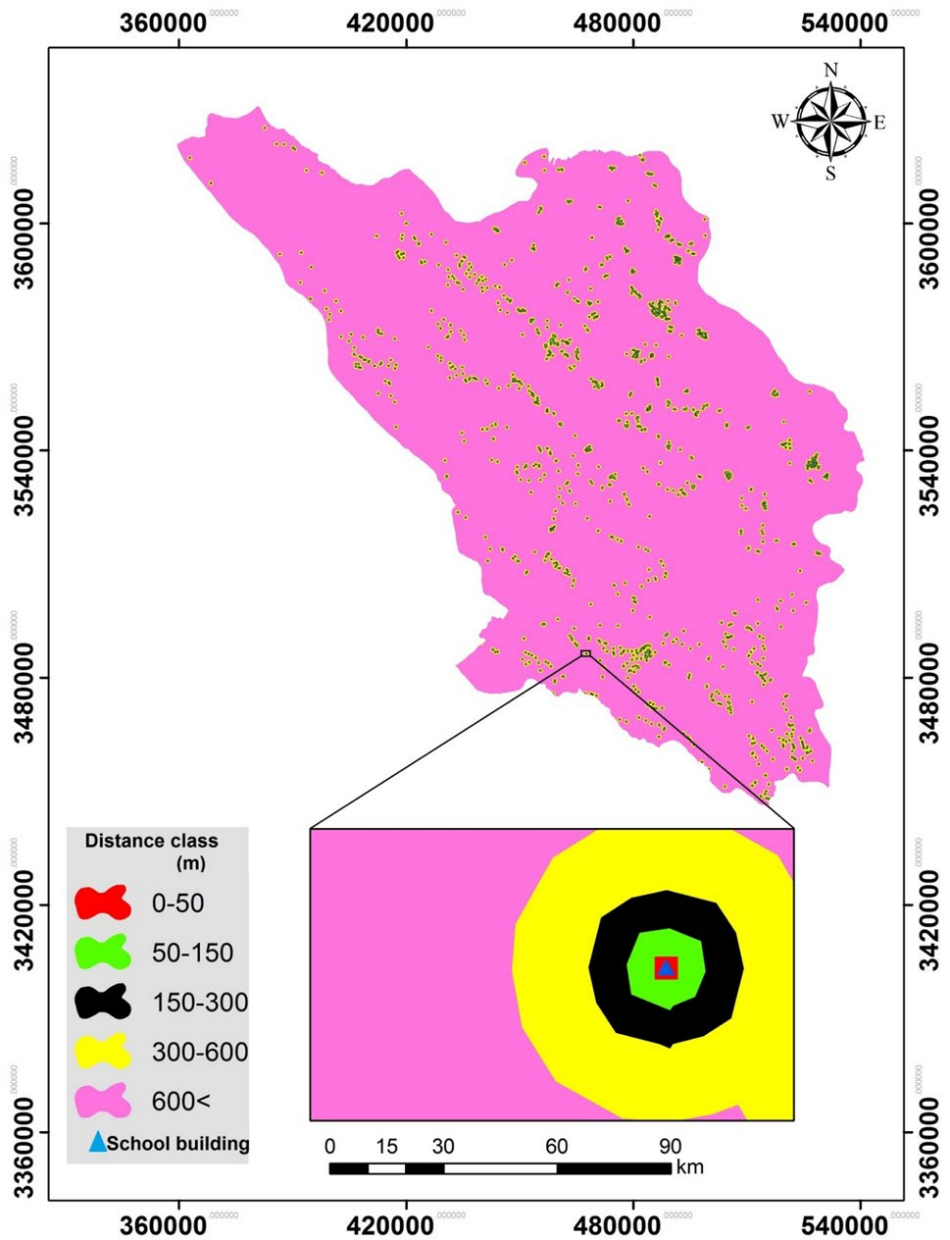
Proximities to schools in Chaharmahal and Bakhtiari Province.

The geolocations of 1,622 school buildings that are attended by 201,274 in Chaharmahal and Bakhtiari Province (Table 1) were identified and mapped. Sixty-three percent of schools were in rural areas and 37% in urbanized areas. Thirty-two experts (hydrologist, educational teachers, fluvial geomorphologists, etc.) completed questionnaires about schools’ distances to flood zones to reflect the exposure of each school to flood hazard. Consistency ratios (CRs) were calculated to evaluate the consistency of the experts’ opinions about school exposures. Arc GIS 10.4.2’s Euclidean-distance tool was used to evaluate the proximity of each school to the modeled flood patterns. Using AHP, distances were classified by concentric rings around school buildings (0–50 m, 50–150 m, 150–300 m, 300–600 m and > 600 m) (Fig. 5). Finally, the normalized rates (NR) of the five distance classes were calculated to determine the weight of exposure for each school.

**Table 1:** Distribution of schools and students in 9 counties.

County	No. of Schools	No. of Students
Ardal	157	12,195
Brojen	214	22,985
Ben	52	5072
Saman	72	5640
Shahrekord	304	48,688
Farsan	135	16,622
Kohrang	164	10,472
Kiar	151	8610
Lordegan	373	70,990
Total	1622	201,274

#### The susceptibility of schools to floods

Based on the natural break algorithm, the flood exposure map was classified into five classes (very low, low, moderate, high and very high) in ArcGIS 10.4.2^15,87^. To generate the final school flood-exposure map, the most accurate flood risk map and school exposure map were fed into the susceptibility equation: Flood susceptibility = Flood risk × School exposure. The susceptibilities of schools in five classes (very low, low, moderate, high and very high) were determined.

## Results

### Flood risk map

**Figure 6:**
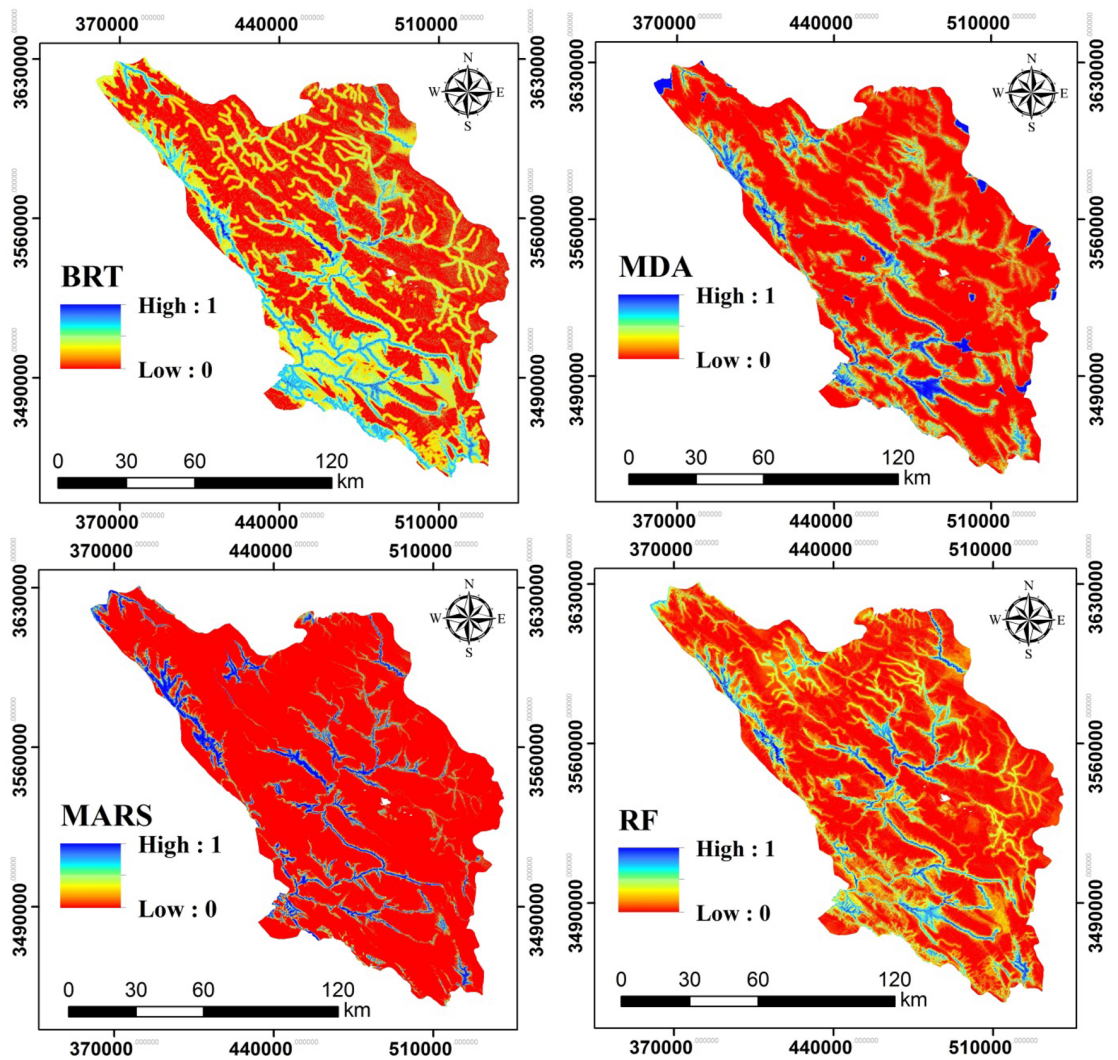
Flood risk maps generated by the four BRT, RF, MARS, and MDA models.

Flood risk maps for Chaharmahal and Bakhtiari Province were produced with BRT, RF, MARS, and MDA algorithms (Fig. 6). The four models generated similar patterns, but they differed in the details of the predictions. The western and southwestern parts of the province are most prone to flood events.

The RF model produced the best flood-risk map (Table 2) by predicting locations that are likely to flood better than the other models. The others, in order of accuracy, were MARS, MDA, and BRT models. But based on AUC analysis, MARS, MDA, and BRT also produced acceptable flood-risk maps (Table 2). The RF model indicates that flood risk in the eastern portion of the study area is much lower than in the central and southern parts of the province.

**Table 2:** Results of model evaluation using the AUC metric.

Test result variable(s)	AUC	Standard error	Asymptotic significant	Asymptotic 95% confidence interval	
				Lower bound	Upper bound
RF	0.989	0.006	0.000	0.978	1.000
MDA	0.970	0.010	0.000	0.950	0.990
MARS	0.978	0.010	0.000	0.959	0.997
BRT	0.957	0.013	0.000	0.931	0.983

### School-exposure map

**Figure 7:**
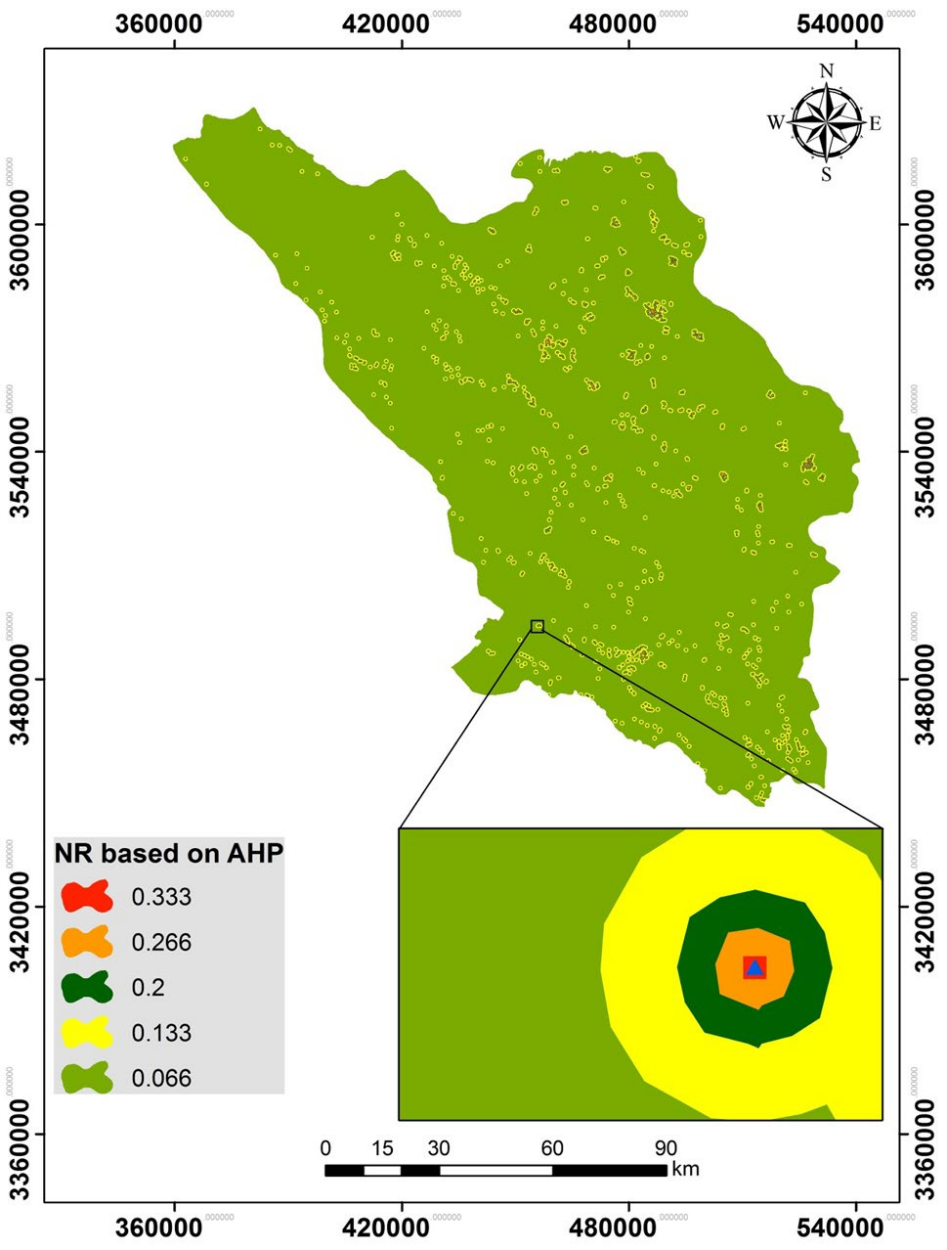
Normalized school-exposure rates based on AHP.

Using AHP, the normalized rate (NR) of the five distance classes were determined (Table 3). The exposure map was prepared according to experts’ ratings for different school-vulnerability classes (based on distance from flood hazard zone)^15,88–90^ and the AHP results (Fig. 7). A consistency ratio (CR) of 0.08 is an acceptable value.

**Table 3:** Normalized rates of school-exposure classes.

Distance from school (m)	Exposure class	Rate	NR
0–50	Very high	9	0.345 (10/29)
50–150	High	8	0.276 (8/29)
150–300	Moderate	6	0.207 (6/29)
300–600	Low	4	0.138 (4/29)
600 <	Very low	2	0.069 (2/29)
Total	–	29	1 (29/29)
Consistency ratio = 0.08

### Susceptibility map

**Figure 8:**
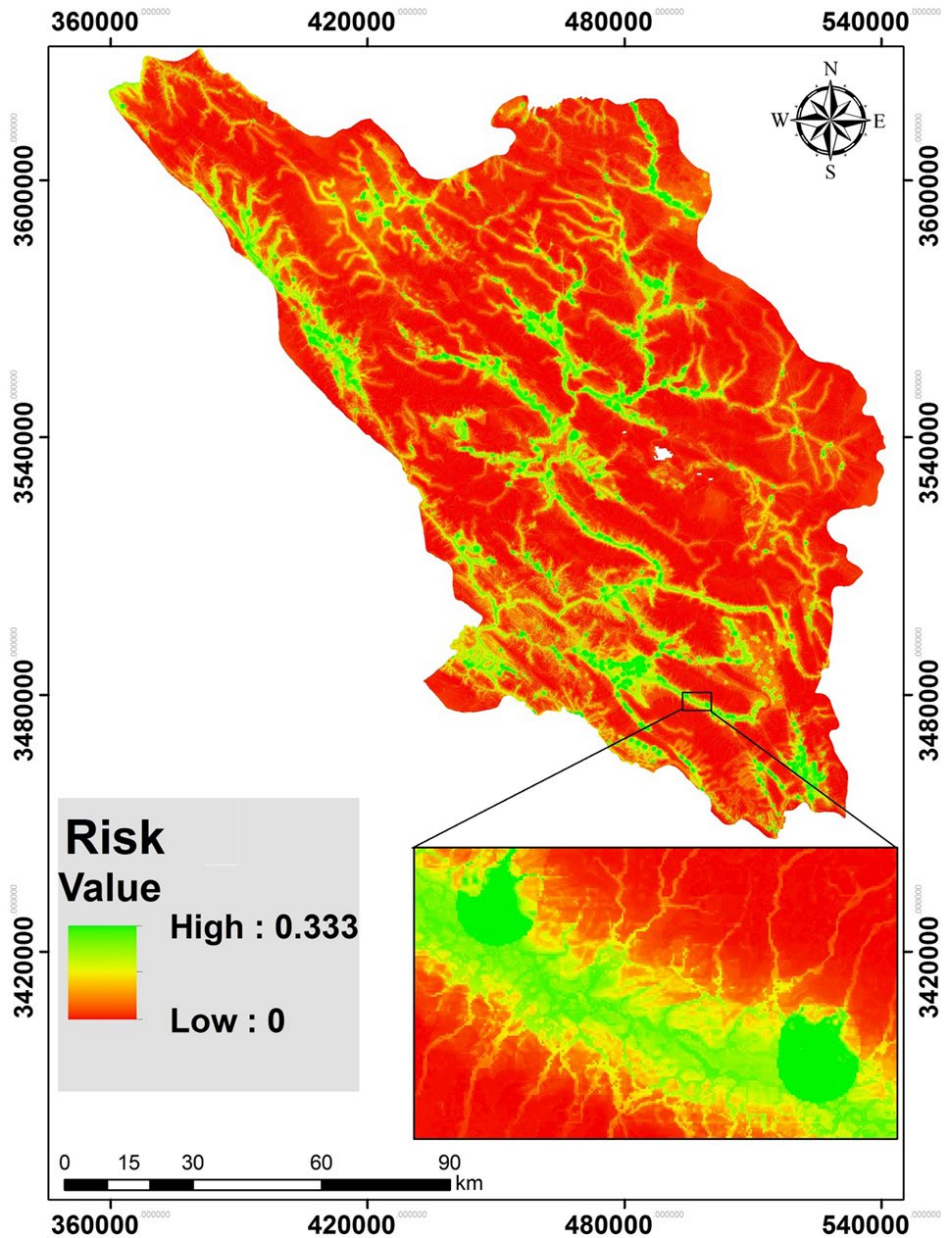
The susceptibility map of schools to flood hazard in study area.

**Figure 9:**
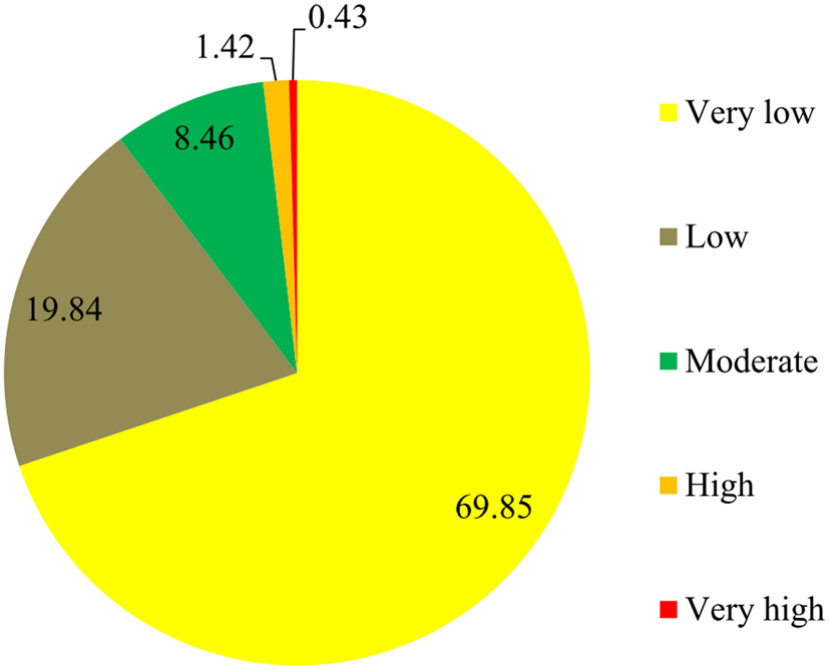
Percentages of the province covered in the five flood-susceptibility classes for schools.

A map of the flood susceptibility of schools (flood risk x exposure of schools) was produced (Fig. 8) using the flood risk modeled using RF. Susceptibility was categorized into five classes based on natural breaks in ArcGIS 10.4.2 (Fig. 9). The results indicate that 69.85% falls into the lowest class of school flood-susceptibility. Only 1.42% of the province has schools that are highly susceptible to flooding and 0.43% has schools in very highly susceptibility circumstances.

### Susceptibility of schools to flood

In all, 979 schools serving 123,324 pupils are in conditions of high and very high flood-susceptibility (Table 4). Of these, 492 are rural schools serving 55,395 pupils and 147 urban schools serving 31,245 students in conditions of very high susceptibility (Table 5). Schools of Lordegan County are the most susceptible to floods: 14,299 pupils in 42 urban schools and 36,312 pupils in 196 rural schools are educated in very high flood-susceptible zones.

**Table 4:** Flood susceptibilities of schools by the accurate model.

Risk class	No. of schools	No. of school class room	No. of students	Average of students per class
Very low	83	617	10,462	17
Low	186	1188	22,684	19
Moderate	374	2341	44,804	19
High	340	1990	36,684	18
Very high	639	3486	86,640	25
Total	1622	9622	201,274	21

**Table 5:** Distribution of schools and students by flood-susceptibility class and county.

County	School type	Very low		Low		Moderate		High		Very high	
		No. of schools	No. of students	No. of schools	No. of students	No. of schools	No. of students	No. of schools	No. of students	No. of schools	No. of students
Ardal	Urban	–	–	–	–	–	–	2	80	18	2239
	Rural	1	4	10	274	37	3646	38	2734	51	3218
Brojen	Urban	18	2513	43	7001	36	4156	36	4556	3	202
	Rural	10	696	22	1483	20	1314	19	969	5	94
Ben	Urban	–	–	4	439	8	915	4	354	4	499
	Rural	7	1097	8	781	11	572	5	394	1	40
Saman	Urban	–	–	1	165	15	2217	1	166	–	–
	Rural	–	–	–	–	5	452	2	118	47	2522
Shahrekord	Urban	25	4511	33	7561	59	10,721	42	8711	52	9704
	Rural	4	634	10	878	21	1596	28	2144	27	2101
Farsan	Urban	2	128	6	778	31	4104	23	3265	19	3481
	Rural	1	21	6	32	5	89	12	1658	31	3191
Kohrang	Urban	3	418	–	–	1	178	7	610	1	4
	Rural	7	185	22	1054	31	2264	26	1070	66	4688
Kiar	Urban	1	159	2	185	22	1996	14	1562	8	818
	Rural	2	11	9	199	7	63	20	409	66	3207
Lordegan	Urban	–	–	–	–	5	1222	5	396	42	14,299
	Rural	2	118	10	1854	60	9299	53	7490	196	36,312

## Discussion

Experts believe that decision makers can reduce losses caused by flood events by implementing mitigation and management actions in watersheds^91–93^. The most important effects of floods are losses of lives, losses of shelter and property, out-migration, disease outbreaks, despair and hopelessness, loss of social capital, and loss of employment. Flood modelling and mapping alone will not reduce hazard and vulnerabilities, but it provides a perspective for mitigation of risk and management of flood hazard in watersheds and in communities. Children are among the most vulnerable in society to hazards and their consequences. As they spend much of their lives in schools, these structures need to be located in places less likely to flood.

**Figure 10:**
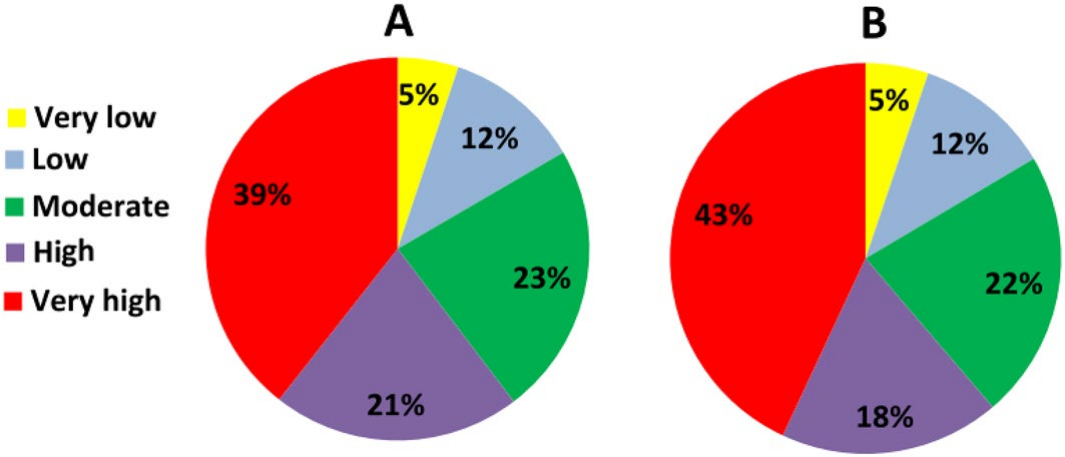
Distribution of schools **(A)** and pupils **(B)** of the Chaharmal and Bakhtiari Province in the five flood-susceptibility classes.

This study assessed the susceptibility of schools to floods in Chaharmahal and Bakhtiari Province. Four ML algorithms (MARS, MDA, BRT and RF) were used to predict the spatial patterns of floods to determine flood risk. The results of validation of the models’ results indicated that RF was the most accurate (AUC = 0.989) of the models. RF uses the most important variables or dividing points within variable subgroups to create a growth tree randomly selected from a set of factors, and thus reduces the importance of each individual regression tree. This shrinks the matching rate, reducing the model error^69^. This method improves the stability and accuracy of the classification, reduces variance, and avoids excessive fitting^67,70^. Finding that the RF model generates an accurate model for prediction and determination of different phenomena is consistent with Taalab et al. (2018), Avand et al., (2019), Hosseinalizadeh et al. (2019), and Gayen et al. (2019). It has been argued that “risk” should be used for flood management as it considers both vulnerability and flood probability simultaneously^25^. The flood risk map allows decision makers to allocate and prioritize places in need of urgent flood mitigation. Based on the flood-risk map produced by the RF model and the school exposure map generated by AHP, the school-susceptibility map was produced (Fig. 10). For vulnerability issues, both quantitative and qualitative datasets were gathered from available reports and through questionnaires and interviews for investigating the different vulnerability dimensions. Social experiences and awareness provide valid information about flood vulnerability^47,96^. There are 1023 and 599 school buildings in the rural and urban parts of the province. Based on the results, 48% (492) of rural schools and 24.5% (147) of urban schools are in conditions of very high flood susceptibility. And 54% (55,395) of students in rural schools and 8% (31,245) in urban schools are in zones of very high school-susceptibility. On the other hand, 76% (297,729) and 2.7% (2733) of children are in very low susceptibility conditions in urban and rural schools, respectively. These results indicate that the rural schools are in more flood-susceptible areas and mitigation of these conditions is urgently needed. This would, most likely, be accomplished by moving schools to less flood-prone locations. Supervision and oversight of the locating, constructing, or reconstructing of schools is usually greater in urban areas than in rural. For this reason, schools and students in urban areas are less susceptible to floods than those in rural areas. The magnitude of challenges for schools and students is expected to grow even further with a population growth, urbanization, and changing climates. Considering the status of schools in terms of flood risk, the current flood defense measures in this province are often unable to cope with additional pressure. As a solution, updated flood risk maps can enhance flood policy and management and can be a rational basis for decision-making.

Susceptibility to floods in the study area should dictate that the 43% of pupils and 39% of schools in very high zones be relocated to safer places. Students are not spared from floods; they suffer losses, too. Damage to school buildings may make them unsafe to the point where they may need to be demolished and rebuilt. The traumas of disasters have been substantiated to impact students' psyches. Shahrekord (home of the province capital) and Brojen counties have 29 and 28 schools, respectively, in zones of very low flood-susceptibility, making them the sub-regions with the safest schools in Chaharmahal and Bakhtiari Province. Historically, villages and cities have been built in flood zones. Moving the buildings and properties to safer locations requires too much money, and is usually socially unacceptable. An alternative is to rebuild schools and public places with stronger, more flood-resistant materials. The cities of Shahrekord and Brojen are the largest and most important in the province, and it is known that governors focus their efforts in these communities to achieve more satisfaction among the residents. Still, there are 9704 pupils in 52 schools in Shahrekord City located in very highly susceptible zones and they remain in serious danger from flooding.

This study described the root causes of flood risk related to schools and provided insight into flood-risk management. Students and children are the future of any country and growing them in a safe environment is essential for any government. This study showed that many of the schools of Chaharmahal and Bakhtiari Province are in worrisome locations, and this concern is even more acute in rural areas. For this reason, it is recommended that safety managers examine the locations of school buildings in their jurisdiction to identify those that are in the most precarious locations. The susceptibility of all future school sites should be carefully considered before they are constructed. All schools should be located in places that are as risk-free as possible. Schools that are at high or very high levels of flood-susceptibility should be relocated to safer places at the earliest possible opportunity, before the next flood disaster occurs. In addition, flood control measures can help to reduce flood risk when building new schools is impractical.

## Conclusion

The susceptibility of schools to floods in the Chaharmahal and Bakhtiari Province, Iran was assessed. Thirty-nine percent of schools are in zones of very high flood susceptibility and urgent action is need by decision makers. Additionally, the susceptibility of rural schools to floods is greater than it is for the schools in urban areas. A total of 86,640 pupils attend schools in locations of very high flood susceptibility in the province. In addition to relocating schools in dangerous places, decision makers should enhance public knowledge and awareness of the threats faced by schools and by children. Results of studies like this one can help raise public awareness, which is an effective soft measure to reduce unavoidable negative impacts of floods. Reducing deaths, damages, and disruptions caused by floods could be facilitated in by education at all levels of society, in schools and publicly. Drills and simulations should be held in schools and rural areas to build preparedness for flood events. Assessment of the susceptibility of schools to flood risks in a mountainous region of Iran is but one part of a management to reduce the likelihood that extreme flood events will turn into tragic disasters.
